# Effect of land use and hydrological processes on *Escherichia coli* concentrations in streams of tropical, humid headwater catchments

**DOI:** 10.1038/srep32974

**Published:** 2016-09-08

**Authors:** Emma J. Rochelle-Newall, Olivier Ribolzi, Marion Viguier, Chanthamousone Thammahacksa, Norbert Silvera, Keooudone Latsachack, Rinh Pham Dinh, Piyapong Naporn, Hai Tran Sy, Bounsamay Soulileuth, Nikom Hmaimum, Pem Sisouvanh, Henri Robain, Jean-Louis Janeau, Christian Valentin, Laurie Boithias, Alain Pierret

**Affiliations:** 1 iEES-Paris (IRD, Sorbonne Universités, UPMC Univ Paris 06, CNRS, INRA, UPEC, Université Paris Diderot), CC237, 4 place Jussieu, 75005, Paris, France; 2 Géosciences Environnement Toulouse (GET), UMR 5563 (CNRS-UPS-IRD), 14 avenue Edouard Belin, 31400 Toulouse, France; 3 IRD-iEES-Paris, Department of Agricultural Land Management (DALaM), P.O. Box 4199, Ban Nongviengkham, Xaythany District, Vientiane, Lao PDR; 4 Soils and Fertilizers Research Institute (SFRI), Duc Thang Ward, Bac Tu Liem District, Hanoi, Vietnam; 5 National Park, Wildlife and Plant Conservation Department (NPD), 61 Phaholyotin Road, Ladyao, Chatuchak, Bangkok 10900, Thailand; 6 National University of Laos (NUoL), Faculty of Agriculture, Nabong Campus, Ban Paksap Mai, Xaythany District, Vientiane, Lao PDR; 7 iEES-Paris (IRD, Sorbonne Universités, UPMC Univ Paris 06, CNRS, INRA, UPEC, Université Paris Diderot), Centre IRD France-Nord, 32 avenue Henri Varagnat, 93143 Bondy Cedex, France

## Abstract

Lack of access to clean water and adequate sanitation continues to be a major brake on development. Here we present the results of a 12-month investigation into the dynamics of *Escherichia coli*, a commonly used indicator of faecal contamination in water supplies, in three small, rural catchments in Laos, Thailand and Vietnam. We show that land use and hydrology are major controlling factors of *E. coli* concentrations in streamwater and that the relative importance of these two factors varies between the dry and wet seasons. In all three catchments, the highest concentrations were observed during the wet season when storm events and overland flow were highest. However, smaller peaks of *E. coli* concentration were also observed during the dry season. These latter correspond to periods of intense farming activities and small, episodic rain events. Furthermore, vegetation type, through land use and soil surface crusting, combined with mammalian presence play an important role in determining *E. coli* loads in the streams. Finally, sampling during stormflow revealed the importance of having appropriate sampling protocols if information on maximum contamination levels is required as grab sampling at a fixed time step may miss important peaks in *E. coli* numbers.

Although much is known about the sources, transport and fate of faecal indicator bacteria (FIB) in temperate climates, a more detailed investigation of the factors influencing the environmental attenuation of FIB in tropical environments is needed^1^. In developing countries river water subject to wastewater contamination is often used for domestic purposes (cooking, washing, etc.). In Asia, for example, it is estimated that over 40% of rural drinking water sources are contaminated^2^. Moreover, many developing countries are experiencing strong demographic pressures along with rapid shifts in land use^3^. In order to respond to the increasing demand for clean water and sanitation, it is essential that we have an understanding of the sources and the drivers of contaminants in tropical aquatic systems. Therefore, recognizing and understanding the relative influence of natural and human-induced processes on hydrological and biogeochemical functioning are prerequisites for improved water resources management.

The microbiological quality of rivers is primarily controlled by human and animal density and the management of their waste in the watershed^4^,^5^. In developing countries and particularly in rural areas, agriculture is less intensive than in developed countries, wastewater treatment is often lacking and diffuse (non-point) sources of FIB tend to predominate^6^. In these rural areas with low human population densities, the primary source of FIB is faecal matter generated by livestock and wild animals. Causse *et al*.^4^, working in rural Laos, found that numbers of *Escherichia coli*, a commonly used FIB^7^,^8^, were below 1 most probable number (MPN) 100 mL^−1^ in the upper areas of the watershed. These authors proposed that this very low background level of contamination was probably caused by wildlife. They also showed that FIB concentration increased as the density of poultry and humans settlements increased in downstream areas.

Other, non-agricultural diffuse sources of microbial pollution in rural watersheds include failing septic systems and latrine overflows during periods of heavy rain. As a consequence of their dispersion, these diffuse sources of microbial pollution are inherently more difficult to identify and characterize than point sources. Moreover, the lack of infrastructure in the rural areas of developing countries also means that very little data is available on FIB contamination. This is particularly so in South East Asia where little information is published in the international literature.

Heavy rainfall and stormwater discharges can rapidly deteriorate surface water quality^9^ by increasing turbidity, suspended solids, organic matter and faecal contamination^4^,^10^,^11^. In urban areas without adequate wastewater treatment or stormflow mitigation management, heavy rainfall washes off FIB and other contaminants from the ground surface^12^. In rural areas, especially in areas with bare soil and/or annual crops, similar heavy rainfall leads to the erosion of soil and, hence of any faecal matter on the soil surface^3^,^13^. This elevated overland flow is due, in part, to higher rates of soil surface crusting under bare soils and in annual and perennial crops (e.g. teak trees) that reduce soil infiltrability^14^,^15^,^16^. Thus in both rural and urban areas, one of the major pathways via which faecal contaminants enter waterways is overland flow^4^,^17^,^18^.

FIB concentrations in storm-generated overland flow are controlled by many factors such as rainfall duration and intensity, manure application, faecal deposit age and type, adsorption to soil particles^1^,^19^,^20^,^21^. Causse *et al*.^4^ reported values of 230,000 MPN 100 mL^−1^ of *E. coli* in overland flow during a storm downstream of a village area. Other authors have also observed higher FIB numbers during storms^10^,^11^ relative to during baseflow. On the other hand, increases in discharge during stormflow may dilute FIB concentrations and differences in land use and vegetation cover may also interact to reduce the transport of FIB from the soil surface into the stream. However, given the high variability in vegetation cover and rainfall that occurs across different study sites and the paucity of data on FIB in rural, tropical catchments, it is difficult to make any regional generalizations at present.

The study presented here was conducted in a network of well characterized (soil, morphology, land use, etc.) catchments. The main hydrological variables (stream discharge), water quality variables (soil loss due to the land erosion, suspended load and bed load), and meteorological variables (air temperature, rainfall, etc.) have been monitored since 2001 in each of the catchments. Given the strong relationships between mammalian presence and *E. coli* loads in streams that have been observed in temperate environments and that the highest overland flows are observed in areas with bare soils and/or crops, we hypothesized that the highest *E. coli* loads would be observed in catchments with high mammalian presence, highest percentage of annual crop cover and during stormflow.

The objectives of this work were (1) to examine the seasonality in FIB loads in three comparable rural catchments located in three countries of South East Asia (Laos, Thailand and Vietnam), and (2) to identify during which hydrological periods (e.g. stormflow periods or baseflow periods) the highest FIB contamination levels are observed.

## Material and Methods

### Study sites

**Figure 1:**
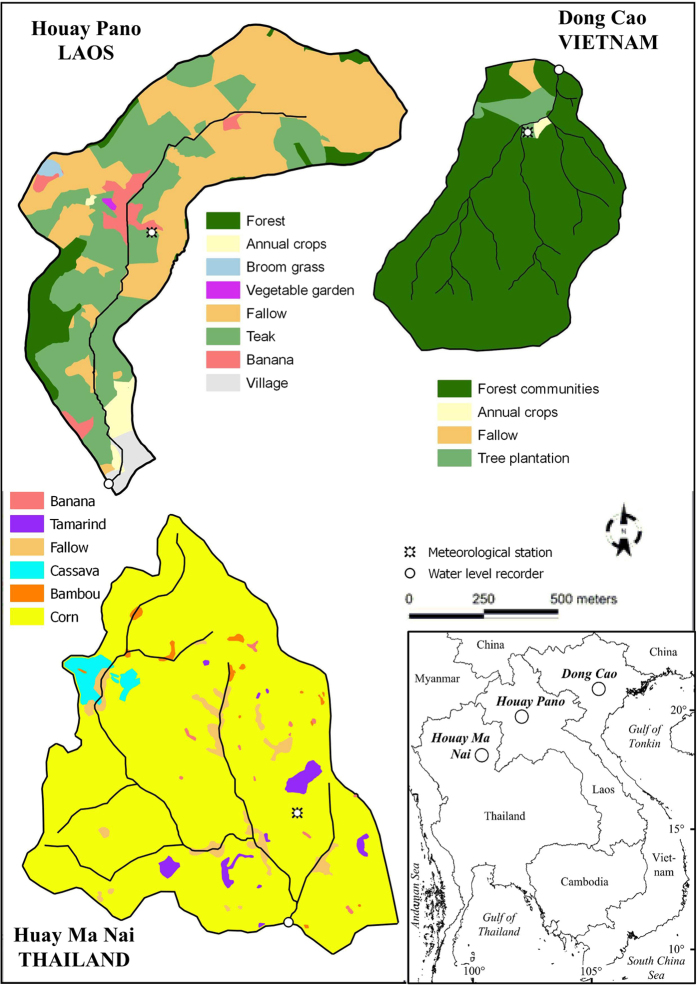
The three studied catchments of the MSEC network and their land use in 2015. The land use maps were created with land survey data from the three sites using QGIS software (http://www.qgis.org/en/site/) and the map of Southeast Asia was created for this article with Microsoft Office Visio 2007.

The three study sites (Fig. 1) are subject to a tropical climate which is influenced by the southwest monsoon bringing warm and humid air masses during the wet season (April-September), and by the northeast monsoon bringing colder, dryer air during the dry season (October-March). Rainfall is highly seasonal with more than 80% of annual rainfall occurring during the wet season. In Laos and Thailand average daily temperatures are highest in April at the end of the dry season when they can reach 40 °C. In Vietnam the highest temperatures generally occur in July and August. Vietnam also has the highest amplitude in terms of temperature variations over the annual cycle (+/−23 °C relative to +/−16 and 15 °C for Laos and Thailand, respectively). Global radiation (GR) varies between 401 and 2,151 J cm^−2^ and GR is the highest in Laos, the lowest in Vietnam and intermediate in Thailand (Table 1).

The three studied catchments are part of the MSEC monitoring network (http://msec.obs-mip.fr/). Each catchment is located in an upland rural area and at a similar elevation range. The catchments were selected for the regional representativeness of their geomorphic, climatic and land use characteristics, for their relatively easy access for field sampling and monitoring, and for their contrasted land uses (Table 1). The Houay Pano catchment is located about 10 km south of Luang Prabang city, Laos. Similar to other parts of Northern Laos, teak tree plantations cover a large part of the catchment surface area (Fig. 1)^18^,^22^. There are some vegetable crops but this is concentrated in small patches along the stream. Otherwise agriculture has remained low input and manual^23^. The Dong Cao catchment is located in Northern Vietnam, about 50 km southwest of Hanoi. As a consequence of soil exhaustion and erosion, declining yields and governmental incentives, tree plantations (e.g. *Acacia mangium*) have increased in extent and density on the steep slopes^18^,^24^. Livestock is also bred in the catchment and non-cultivated land is used for pasture or is partly planted with grass fodder (*Bracharia ruziziensis*)^25^. The Huay Ma Nai catchment is located in Northern Thailand, about 550 km north of Bangkok. Maize is the dominant crop and agriculture in the catchment is dependent on external inputs of fertilizers and pesticides and on frequent use of machinery for tillage^26^.

### Hydro-meteorological measurements and water sampling

Daily rainfall data was collected by automatic rain gauges (Campbell ARG100, 0.2 mm capacity tipping-buckets) in each of the three catchments. Stream water level was measured at the outlet of each catchment with 1 mm vertical precision at a minimum of 3 minute time interval by a water level recorder (OTT, Thalimedes) equipped with a data logger within a V-notch weir in Laos and Vietnam and a Parshall flume in Thailand. A control rating curve (the relationship between water level and discharge) was determined using the velocity area method at each station. The accuracy of stream discharge (Qsw) is considered to be within +/−10% of the measured value.

Samples of streamwater (500 ml) were collected in new, clean, plastic bottles during base and storm flows approximately twice per month at the outlet of each catchments from October 2014 (end of rainy season 2013–2014) to October 2015 (end of rainy season 2014–2015; Table 1). Temperature and electrical conductivity (EC) of the streamwater were measured using a Multi Probe System (YSI 556 MPS) in Laos, and a WTW multiparameter probe (MultiLine^®^ Multi 3410 IDS) in Thailand and Vietnam. The uncertainty of EC is estimated to be ±5% of stream EC value, which corresponds to the instantaneous variability in the stream section during a storm event.

The concentration of total suspended solid (TSS) was determined for each sample after filtration on 0.2 μm porosity cellulose acetate filters (Sartorius) and evaporation in an oven at 105 °C for 48 h. There was no TSS data available for Huay Ma Nai due to a technical problem. The uncertainty is considered to be within +/−10% of the measured value for TSS.

### *E. coli* determinations

The standardized microplate method (ISO 9308-3) was used for *E. coli* number determinations. Each sample was incubated at four dilution rates (i.e. 1:2, 1:20, 1:200 and 1:2000) in a 96-well microplate (MUG/EC, BIOKAR DIAGNOSTICS) and incubated for 48 h at 44 °C. Ringers’ Lactate solution was used for the dilutions and one plate was used per sample. The number of positive wells for each microplate was noted and the MPN was determined using the Poisson distribution. This microplate method has previously been used with success in one of the studied agro-ecosystems^4^,^9^.

### Soil surface crusting rate

Because soil surface crusting has been found as the most effective predictor of overland flow in these catchments^14^,^16^,^25^,^27^, it was used in this study as a proxy of overland flow. On the scale of the catchment it was determined from data on vegetation cover in each catchment (Fig. 1) and the values of soil surface crusting rates from hundreds of data collected under different land uses and vegetation cover for Houay Pano^16^. The soil surface crusting rate ascribed to each land use and vegetation cover was assumed to be homogenous for each land use type across each of the three catchments.

### Statistical analyses

The significance of the differences between parameters across the three catchments was determined using the program R^28^. The Welch two-sample t-test was used to determine the significance of the differences between crusting rates, runoff rates and people.days. The Kolmogorov-Smirnov test was used to determine if the distribution of the *E. coli* concentrations was significantly different between catchment. Significance was set at p < 0.05.

## Results

### Seasonal variations

#### Houay Pano, Laos

**Figure 2:**
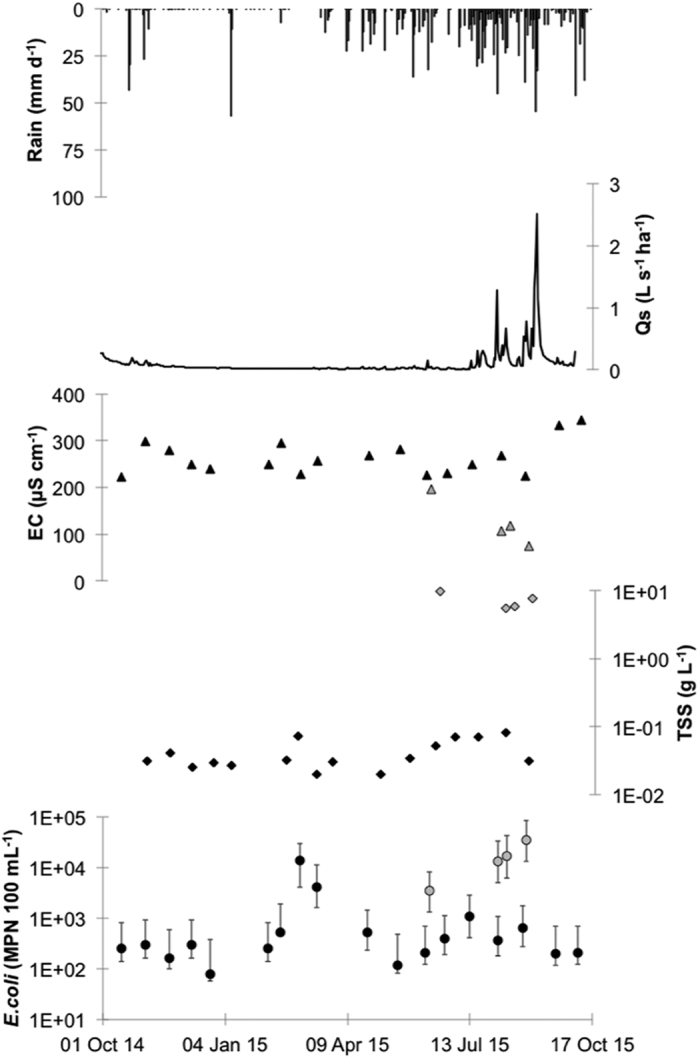
Baseflow (inter-storm) and stormflow in the Houay Pano catchment, Laos. Rainfall intensity (daily time step; mm d^−1^); stream specific discharge (Q_s_, L s^−1^ ha^−1^); electrical conductivity (EC, μS cm^−1^); total suspended solids (TSS, g L^−1^); *E. coli* concentration in stream (*E. coli*, MPN 100 mL^−1^). Black filled symbols: baseflow samples, grey filled symbols: stormflow samples.

Annual rainfall (October 2014 to October 2015) in the Houay Pano catchment, Laos was 1,465 mm and was close to the annual average of 1,585 mm (Table 1). Over the sample year, several rain events occurred that resulted in a rapid increase in discharge (Fig. 2). This was particularly noticeable during the wet season (April to October) where the repetition of rain events of sufficient intensity and volume resulted in a peak of discharge of 2.51 L s^−1^ ha^−1^ on September 4^th^, 2015. At this site, EC varied between 223 and 345 μS cm^−1^ and was the lowest during periods of high rainfall. TSS also varied over the year with higher concentrations observed during the high rainfall periods concomitant with high discharge and low conductivity. *E. coli* concentrations also varied between 78 and 14,000 MPN 100 mL^−1^ and were higher during periods of high discharge.

In Houay Pano, four stormflow samples were measured corresponding to four separate storm events (Fig. 2). For each of these samples, the values of EC, TSS and *E. coli* differed considerably from baseflow values. For example, EC varied between 74 and 197 μS cm^−1^ as compared to an average of 264 μS cm^−1^ for the non-storm event samples. Moreover, TSS and *E. coli* concentrations were over two orders of magnitude higher in the stormflow samples as compared to during the baseflow period.

#### Dong Cao, Vietnam

**Figure 3:**
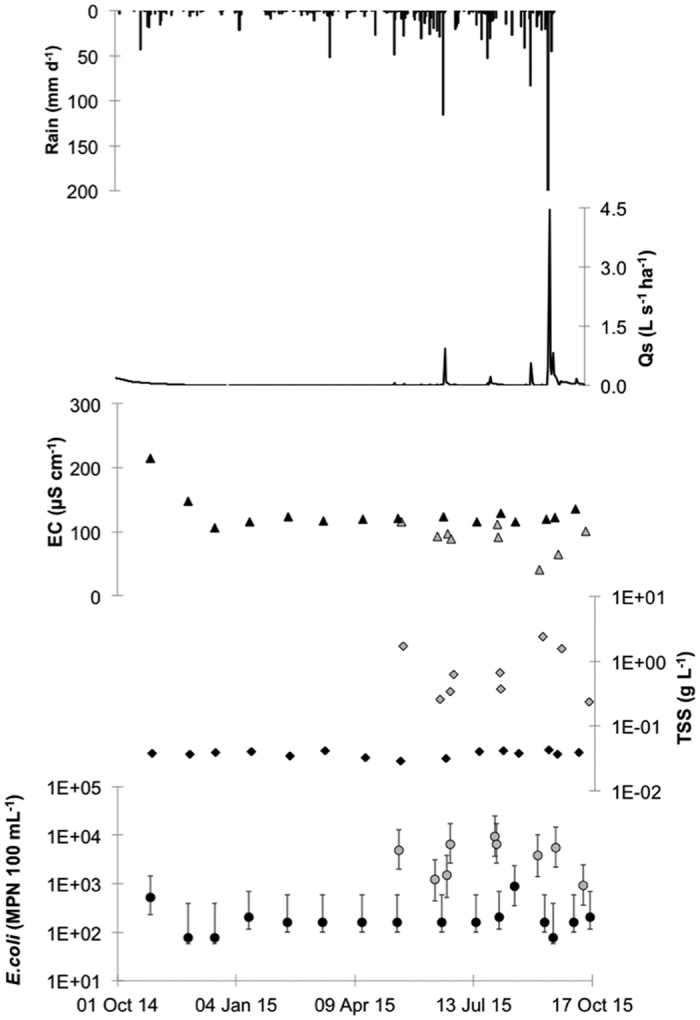
Baseflow (inter-storm) and stormflow in the Dong Cao catchment, Vietnam. Rainfall intensity (daily time step; mm d^−1^); stream specific discharge (Q_s_, L s^−1^ ha^−1^); electrical conductivity (EC, μS cm^−1^); total suspended solids (TSS, g L^−1^); *E. coli* concentration in the stream (*E. coli*, MPN 100 mL^−1^). Black filled symbols: baseflow samples, grey filled symbols: stormflow samples.

A similar pattern was observed in Dong Cao, Vietnam (Fig. 3) although rain intensity was higher in Vietnam during the study period, reaching 209 mm d^−1^ on September 17^th^, 2015. Total annual rainfall the catchment was 1,474 mm, close to the annual average of 1,556 mm (Table 1). As in Laos, heavy rains resulted in peaks of discharge in the stream and the highest discharge (4.55 L s^−1^ ha^−1^) was observed on September 17^th^, 2015 concomitant with the highest rainfall intensities. EC decreased initially from October 2014 to December 2014 and thereafter remained relatively stable over the year. A similar pattern was observed for TSS. *E. coli* numbers varied from 78 to 890 MPN 100 mL^−1^ and were significantly lower (p < 0.05) than those observed in Laos. The highest numbers found during the periods of higher rainfall. Compared to the Houay Pano catchment, the differences in values between baseflow and stormflow in the Dong Cao catchment were lower. For example, EC was about 50 μS cm^−1^ lower in the stormflow samples, relative to EC during the baseflow period. Moreover, *E. coli* numbers were only an order of magnitude higher in the stormflow samples whereas TSS was two orders of magnitude higher.

**Figure 4:**
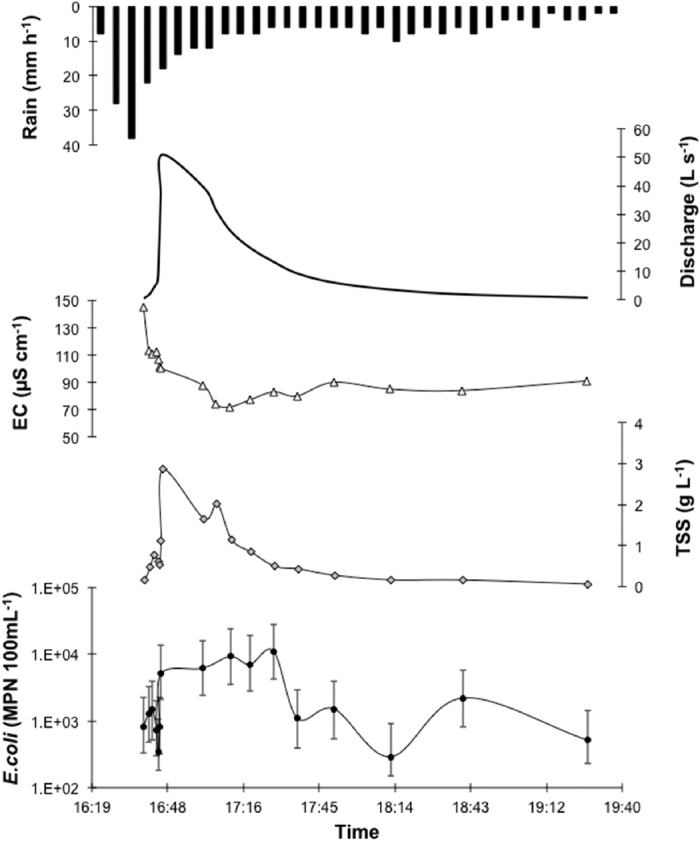
Stormflow event in the Dong Cao catchment, Vietnam. Rainfall intensity (hourly time step; mm h^−1^); stream discharge (Q, L s^−1^); electrical conductivity (EC, μS cm^−1^); total suspended solids (TSS, g L^−1^); *E. coli* concentration in the stream (*E. coli*. MPN 100 mL^−1^).

A detailed sampling was also conducted during a short duration storm event (169 minutes duration) in this catchment (Fig. 4). On the 6^th^ June 2015 a rain event with a peak intensity of 40 mm h^−1^ occurred at 16:45. This resulted in rapid increase in stream discharge that peaked at 51 L s^−1^. The peak in discharge was followed by a decrease in EC from 150 μS cm^−1^ to 65 μS cm^−1^. The decrease in EC was also paralleled by an increase in TSS, the dynamic of which closely followed that of discharge. Over the course of the storm event, *E. coli* numbers varied between 290 to over 11,000 MPN 100 mL^−1^, a factor almost 40 increase. *E. coli* numbers then decreased with the declining flood phase to reach values similar to initial (pre-flood) values of 510 MPN 100 mL^−1^.

#### Huay Ma Nai, Thailand

**Figure 5:**
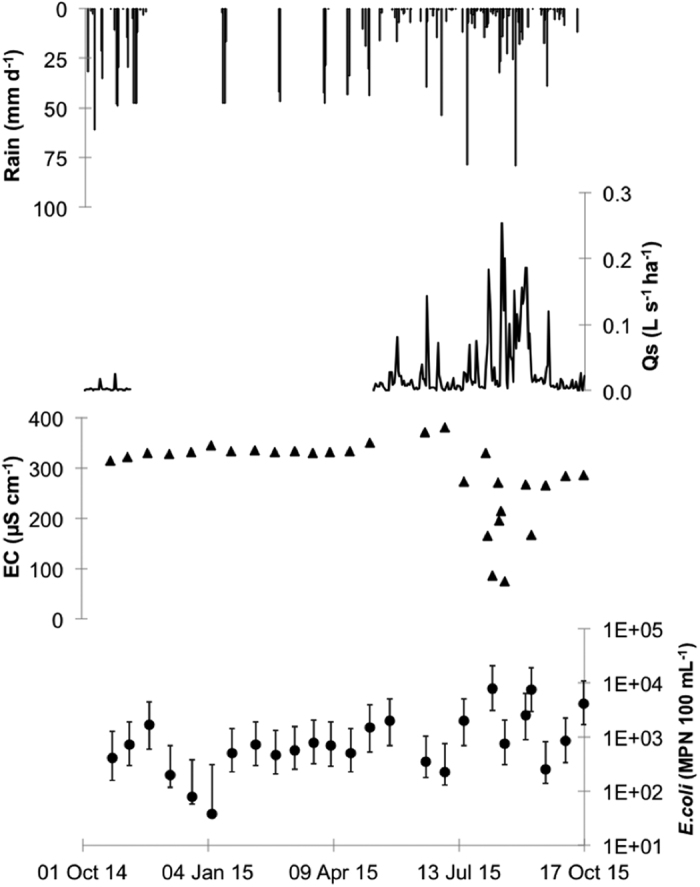
Baseflow (inter-storm) and stormflow in the Huay Ma Nai catchment, Thailand. Rainfall intensity (daily time step; mm d^−1^); stream specific discharge (Q_s_, L s^−1^ ha^−1^); electrical conductivity (EC, μS cm^−1^); total suspended solids (TSS, g L^−1^); *E. coli* concentration in the stream (*E. coli*, MPN 100 mL^−1^). Black filled symbols: baseflow samples, grey filled symbols: stormflow samples.

**Figure 6:**
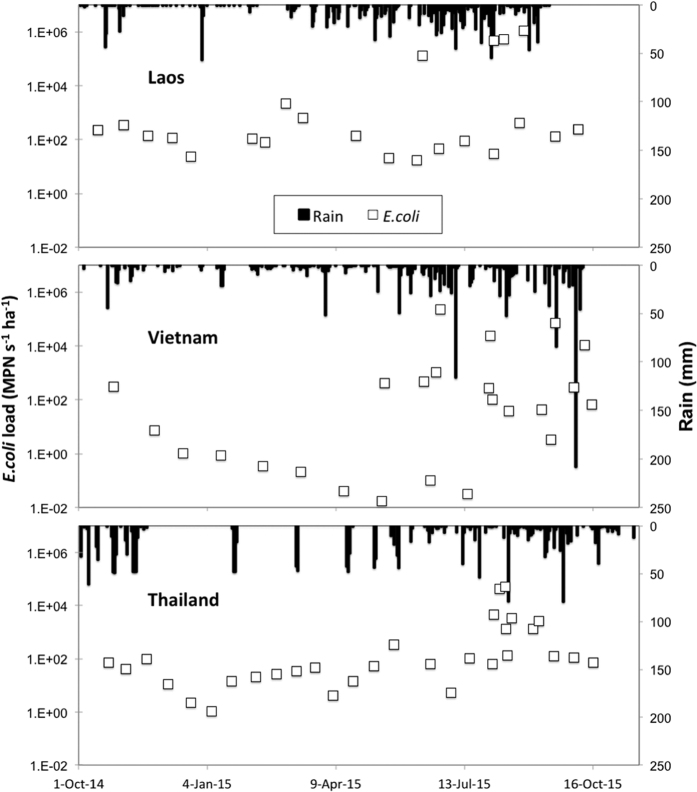
*E. coli* loads and rainfall for each of the three studied catchments (*E. coli*, MPN s^−1^ ha^−1^).

In contrast to Houay Pano and Dong Cao, annual rainfall over the study period in the Huay Ma Nai catchment in Thailand was over 30% lower than the annual average (921 mm versus 1,385 mm; Table 1). Nevertheless, as for the Houay Pano and Dong Cao catchments, episodes of heavy rainfall resulted in an increase in stream discharge (Fig. 5). Indeed, discharges of up to almost 0.25 L s^−1^ ha^−1^ were observed on the August 14^th^, 2015. Large decreases in EC were also observed during the periods of high discharge. Indeed, EC decreased from 320 μS cm^−1^ during the periods of low discharge to 70 μS cm^−1^ during the period of high discharge (3^rd^ August–12^th^ September 2015). *E. coli* concentrations also varied over the year and were of the same order of magnitude as for Houay Pano (78 and 14,000 MPN 100 mL^−1^; p > 0.05). They were however, significantly higher than the concentrations observed in the Vietnamese catchment (p < 0.05). As in the other catchments, the highest values were observed during the summer months when rainfall and discharge were highest. The loads of *E. coli* for each catchment over the sample year are shown in Fig. 6. As for the concentrations, loads were lowest during the dry season when rainfall and discharge was lowest. A significant difference was observed between catchments for *E. coli* loads during dry season (from October 2014 to mid May 2015).

#### Hydrology and land use

**Figure 7:**
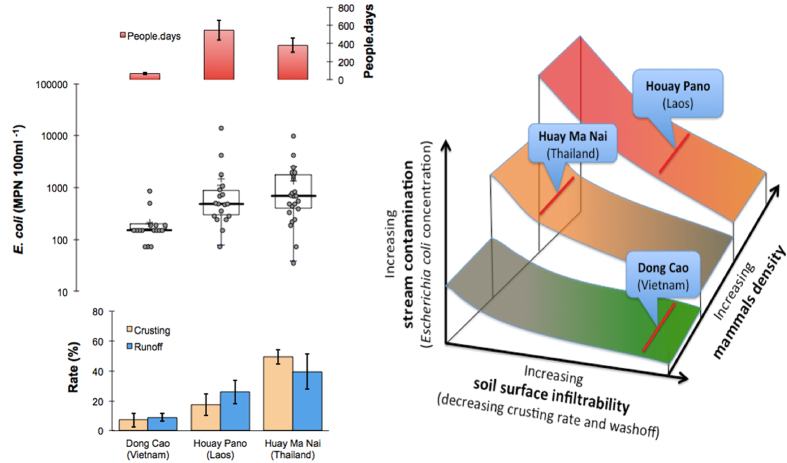
*Escherichia coli* (*E. coli*), soil surface infiltrability and mammalian presence in the studied catchments. Concentrations of *E. coli* in streamwater during baseflow (Dong Cao ≠ Houay Pano = Huay Ma Nai; p < 0.05), soil surface crusting rate (Dong Cao < Houay Pano < Huay Ma Nai; p < 0.05), runoff (Dong Cao < Houay Pano < Huay Ma Nai; p < 0.05), and people.days (Dong Cao < Huay Ma Nai < Houay Pano; p < 0.05) as a proxy of mammalian density (left side panel). Conceptual diagram showing the relationship between mammalian density, soil surface infiltrability and stream *E. coli* loads (right side panel). Each of the three studied catchments is placed on the graph as indicated by a red line. A change in either soil infiltrability or mammalian density would result in a change in the position along and across the different zones.

In Dong Cao, where *E. coli* concentrations, human and animal presence and soil surface crusting^16^,^25^ (vegetation cover is almost exclusively *Acacia mangium*) in the watershed was significantly lower than in the two other catchments (p < 0.05; Fig. 7). This site is also characterized by low erosion rates and low overland flow (excepting the tropical storm on 17^th^ September 2015). In contrast, in Huay Ma Nai, maize is the predominant crop, soil crusting is high^16^,^27^ and up to 150 people can be in the catchment during specific periods. This situation results in significantly higher overland flow (p < 0.05) and significantly different *E. coli* numbers in the stream (p < 0.05). In between these two situations, the Houay Pano catchment is intermediate. Vegetation cover is more mixed, with teak tree plantations and fallow being the most prevalent. This mixed situation gives strongly contrasting soil crusting rates with very high rates in the teak tree plantations and very low rates of soil crusting in the fallow areas^16^. This results in an intermediate soil crusting rate on the scale of the catchment. However, Houay Pano also has a high number of people.days. These two factors (soil surface crusting and human presence) interact to give streamwater *E. coli* concentrations that are significantly different from those of Dong Cao (p < 0.05) but similar to those of Huay Ma Nai (p > 0.05, Fig. 7). Moreover, the differences between Dong Cao and Houay Pano are all the more evident during the storm events when overland flow is higher than during baseflow (Figs 2 and 3).

## Discussion

### Seasonality

The three study sites are subject to strong seasonal differences. Indeed, in Houay Pano, Laos, *E. coli* concentrations were over two orders of magnitude higher during the wet season than during the dry season. Although the difference in *E. coli* concentrations between the two seasons was lower in the other two sites, there was at least one order of magnitude difference between the dry and the wet seasons. This marked seasonal difference in FIB numbers has been previously observed^29^,^30^ and the few data that exist from humid tropical regions also confirm this trend^4^,^6^. The large differences in FIB numbers between the two seasons are principally due to two factors. Firstly, FIB in streams in rural areas, such as in the catchments examined in this work, originate from human and animal defecation^4^. In some areas unrestricted access to waterways can result in the direct inoculation of the watercourse with FIB^31^, however, in these catchments defecation mostly occurs on the slopes in grazing areas, in areas with latrines or in informal defecation sites^4^. One can therefore hypothesize that little or no transfer of faecal matter into the waterways occurs during the dry season, although it should be borne in mind that humans and animals can cause the local resuspension of soil or sediment particles when they use or cross the stream during dry periods^10^. In contrast, during periods of rainfall that induce overland flow, large quantities of faecal matter are washed off into the stream from the soil surface in the catchment^4^,^32^. This explains why much higher FIB numbers are found in the stream during the wet season.

This explanation is rather simplified and does not take into account the resuspension of bacteria during storm events when stream flow is high. It has been shown that the resuspension of particle-associated FIB can be an important source of FIB to the water column during flood events^9^. Nor does it take into account the impact of groundwater flow on *E. coli* numbers. Groundwater generally has very low concentrations of FIB compared to overland flow^33^,^34^ and when it mixes with overland flow, the streamwater is diluted, thereby decreasing the concentration of FIB. This dilution can be particularly high in systems where the contribution of groundwater flow to stream flow is high. However, while groundwater itself dilutes streamwater, at least in terms of *E. coli* numbers, high groundwater fractions are often accompanied by a strong shear stress, which stimulates streambed sediment resuspension resulting in higher *E. coli* numbers. This resuspension can represent a significant secondary source of FIB^1^,^9^.

#### Land use, hydrology and potential health threat

The variability in human activity over the annual cycle in the three catchments also influences *E. coli* concentrations in the streams (Fig. 7). In Thailand, maize crops are harvested during September and October at the end of the rainy season. This is a labour intensive activity involving many people in the field (up to 150 at a time). During this period, informal latrines are used. This probably results in injections of faecal material into the stream as evidenced by the increased *E. coli* numbers in the stream during this period (Fig. 5).

A similar situation occurs in Houay Pano, Laos. In general, preparation of the fields for the growing season starts in March and April. During this period, the use of informal latrine sites increases. Due to a lack of sanitation facilities in the upper catchment, people defecate out of sight in fallow plots along hillslopes or in the vicinity of the stream in the remaining riparian vegetation. At this time of year, small rain events are frequent resulting in short episodes of overland flow and increased discharge. These small events are characterized by highly contaminated overland flow^9^ and this probably explains the very high *E. coli* numbers and TSS observed in the Houay Pano stream in March (Figs 2 and 7). March and April are the hottest months of the year in Laos and streamflow is lowest. During these months, farmers and villagers often use streamwater for their domestic requirements as many wells become dry at this time of year (personal observation). Moreover, in the upper sections of the catchment where most of the crops are grown (Fig. 1) there are no potable water sources and the farmers drink streamwater and water from ephemeral unprotected wells that can be easily contaminated by overland flow.

Clearly, the consumption of contaminated stream water holds some health risks^35^. Indeed, for many of the sample dates, *E. coli* numbers exceeded the limit of 500 colonies 100 mL^−1^ above which the World Health Organization considers that there is a 10% risk of gastro-intestinal illness after one single exposure^36^. Therefore, the use of water from sites with high FIB numbers poses a serious threat to public health. We did not conduct epidemiological studies in parallel with this work; however, it would be interesting in future studies to investigate this health threat.

#### Methodological aspects

The differences in FIB numbers between baseflow and stormflow samples of up to two orders of magnitude also highlight the importance of having appropriate sampling protocols. If grab samples are taken at a fixed time step then an average estimate of FIB numbers will be obtained which may miss any peaks due to short, rapid events. The rain events in the catchments investigated here resulted in flash floods with lag times on the order of 10 to 15 minutes^9^ and with peaks of contamination of 14,000 MPN 100 mL^−1^. These “hot moments”^37^ of contamination would have been missed if samples had not been collected during stormflow. This is the case in all three catchments as presented here as well as in city sewer drains in tropical Singapore^11^ and in other work^32^,^35^ further highlighting the importance of adequate sampling during stormflow. The selection of sampling frequency will depend on the objectives of the study nevertheless if an estimation of the variability of FIB numbers is required, it is probably better to select a variable time step that is linked to discharge rather than to a fixed time step. By fixing the sample interval to an increase in discharge, one can sample during events with significant overland flow and sample “hot moments” with a high frequency. Moreover, having information on *E. coli* concentrations over a much longer period of time (e.g. 10 years) would enhance the robustness of the conclusions of this type of study. However, such a long term program would also need to be considered in the context of the social, economic and technological development trajectories of the studied countries.

An aspect that was not investigated in this work is the possibility of regrowth of *E. coli* in the stream itself^1^. Several authors have proposed that *E. coli* can grow in the environment, i.e. outside their original host organism^38^,^39^. This could be particularly problematic in humid, tropical countries such as those investigated in this work as temperatures remain high throughout the year. The presence of small swamps and ponds along the stream course in these small catchments may also provide an ideal environment for the naturalization of *E. coli*. Small ponds are often used for aquaculture and they are sometimes also fertilized with manure, thereby increasing the *E. coli* available nutrients and hence the numbers of *E. coli*^20^. These ponds are often shallow, but due to riparian vegetation, light penetration is low. Thus, the high concentrations of organic matter and nutrients present may provide an adequate nutritional source to *E. coli* and the shallow sediments provide a physical protection against solar light and predators. All of this contributes to favour the growth of *E. coli*^1^ in the environment. Finally, the application of more advanced techniques such as the identification of biomarkers of faecal matter sources^40^,^41^ provides an interesting way of identifying FIB sources (e.g. chickens, pigs, humans), particularly in these mixed use catchments. Knowing the sources of FIB also helps to evaluate the level of risk associated with the contamination. However, the analytical capacities in the study sites were insufficient; it is therefore difficult to determine if the faecal contaminants are of human or animal origin.

## Conclusions

We show that land use, vegetation cover and hydrology, strongly control *E. coli* concentrations in three tropical, rural streams in Southeast Asia confirming what has been previously shown for temperate environments^42^,^43^ and for pathogenic bacteria in this region^44^. The maintenance of adequate vegetation and the use of management practices that reduce erosion will not only reduce soil losses and increase soil carbon storage, it will also contribute to better surface water quality thereby reducing the risks to the human populations using the water resource^45^,^46^. In future work, it will be necessary to apply modelling techniques that are adapted to these small, montane water courses to understand and quantify the different roles of overland flow and groundwater flow during flood events. This is particularly important as regards the opposing roles that groundwater outflow may have on streamwater contamination during stormflow events: 1) the dilution effect of contaminated overland flow waters along the stream path, and 2) the resuspension of contaminated sediments in the streambed. Having more fine scale data on the dynamics of the different water masses and their relative *E. coli* concentrations during storm events is a fundamental requirement for these models. The relatively high levels of background contamination in each of the three watersheds points towards the need for more information on the behaviour of FIB in tropical environments. Better estimates of FIB die-off rates in these rural, montane watersheds and a clarification of whether or not these indicator bacteria are capable of surviving and growing outside their host are necessary.

## Additional Information

**How to cite this article**: Rochelle-Newall, E. J. *et al*. Effect of land use and hydrological processes on *Escherichia coli* loads in streams of tropical, humid headwater catchments. *Sci. Rep*. **6**, 32974; doi: 10.1038/srep32974 (2016).

## Figures and Tables

**Table 1:** Study sites and catchments characteristics.

Country	Laos	Thailand	Vietnam
Catchment name	Houay Pano	Huay Ma Nai	Dong Cao
Province	Luang Prabang	Phrae	Hoa Binh
Latitude	19°51′10′′N	18°13′20′′N	20°57′40′′N
Longitude	102°10′45′′E	100°23′40′′E	105°29′10′′E
Catchment size	60.2 ha	93.0 ha	49.7 ha
Elevation range	430–718 m	400–480 m	130–482 m
Mean slope	48%	30%	40%
Mean annual rainfall	1585 mm	1385 mm	1556 mm
Rainfall during 2015	1465 mm	921 mm	1474 mm
Mean annual temperature	25 °C	27 °C	23 °C
Mean annual global irradiance	1385 J cm^−^^2^	751 J cm^−^^2^	340 J cm^−^^2^
Geology	Shale, schist	Siltstone, sandstone	Schist
Soils	Alfisol, Entisol Ultisol	Alfisol, Ultisol	Ultisol
Population (number of people.days)^1^	No permanent population, many daily activities (548 +/− 120)	No permanent population, up to 80 during planting and harvest time (380 +/− 72)	A household of 4 with a toilet for which the overflow empties into the stream, 3 farmers that go through basin every 3 days (68 +/− 14)
Animal husbandry	Two to three pigs in the lower sections and some (~20) chickens in both the upper and lower sections	Animal husbandry limited to a few chickens	20 cows, 10 buffalos in the upper sections and 20 chickens and 2 or 3 pigs in the lower sections
Crusting rate^2^	17 (+/−7)%	48 (+/−5)%	7 (+/−5)%
Runoff coefficient^3^	26 (+/−8)%	40 (+/−12)%	9 (+/−3)%
Bi-weekly sampling dates	15 October 2014 to 5 October 2015	23 October 2014 to 16 October 2015	27 October 2014 to 15 October 2015

A short description of the human population in each catchment is given as is an estimate of the people.days which gives an estimate of the number of whole days a person is in the catchment.

^1^Estimated from the number of people using informal latrines in the catchment on a daily basis.

^2^Calculated from ref. 16 and from land use for 2015.

^3^Calculated over the four wettest months of the year (June to September) using streamflow depth ÷ rainfall depth and expressed as a percentage.
